# A 13-Year Long-Term Follow-Up of a Case Report With Continued Improvement in Severe Chronic Neck and Head Pain Alleviated With Chiropractic BioPhysics® Spinal Rehabilitation Protocols

**DOI:** 10.7759/cureus.59024

**Published:** 2024-04-25

**Authors:** Jason W Haas, Robert H Berry, Paul A Oakley, Deed E Harrison

**Affiliations:** 1 Research, CBP NonProfit, Inc., Windsor, USA; 2 Research, CBP Non-Profit, Inc, Montour Falls, USA; 3 Kinesiology and Health Science, York University, Toronto, CAN; 4 Chiropractic, CBP Non-Profit, Inc, Newmarket, CAN; 5 Research, CBP NonProfit, Inc, Eagle, USA

**Keywords:** long-term follow-up, conservative, cbp rehabilitation, chronic neck pain, spine trauma

## Abstract

Alleviation of headaches (HAs), neck pain (NP), and disability is a desirable clinical outcome for the billions globally who suffer from these conditions. Chiropractic BioPhysics® (CBP®) methods may provide an option for head and neck-injured patients. A 62-year-old female historically injured multiple times including two motor vehicle collisions (MVC), and a strike to the face with a hockey puck; all resulting in chronic pain and suffering. The subject sought and received successful treatment in 2016 using this conservative protocol at a facility in the USA. The resolution of symptoms following 36 treatments was previously reported. Following 13 years without treatment beyond home exercises, the subject was re-evaluated and found to be stable in the long term for pain, structural and functional assessment. Thirty-six treatments over 12 weeks in 2016 led to an improvement in numerical pain rating scale (NPRS) for NP (5/10 to 1/10), and HA (9+/10 to 0/10), resolution of NP disability (6/100 to 0/100) as well as normalization of ROM without pain and resumption of all activities of daily living including high-level athletics without pain and disability. A 13-year follow-up found continued stability objectively and subjectively. We provide a case of successful conservative treatment using specific traction, exercises, and spine manipulation procedures. CBP^®^ provides an option to treat pain and this case adds to growing evidence.

## Introduction

The burden of chronic musculoskeletal pain is significant and conservative treatments are desirable for patients and treating physicians globally. We present a case report of successful treatment for chronic neck pain (NP) headaches (HAs), with loss of normal range of motion (ROM) and disability using postural and structural rehabilitation. The patient had significant improvement following a brief period of in-office treatment and reported the NP and HAs resolved with care. Improvements were found in subjective reports, as well as sagittal and coronal radiographs and postural photos. This Chiropractic BioPhysics® (CBP®) treatment protocol has been previously published with positive outcomes for multiple conditions across various populations from injured patients, chronic pain patients, pediatric spine disorders, elderly conditions as well as athletes and asymptomatic patients seeking conservative treatment [1-5].

NP is commonly reported as the fourth leading cause of disability and HA is frequently in the top 10 listed conditions. Given the massive contribution to the global burden of disease (GBD) from NP, HAs, and chronic spine pain and associated conditions, having a successful, non-invasive, economical treatment method that provides not only short-term pain relief and reduction of abnormal health-related quality of life (HRQoL) measures, but also long-term stability and continued improved patient-reported outcomes (PROs) is requisite [6-9]. Improved spine alignment radiography, postural balance as well as reduced pain and disability are consistent with prior reports and clinical trials of CBP® protocols. This case adds to a growing body of evidence of the success of CBP® mirror image (MI®) methods [1-5]. The purpose of this case is to report the 13-year long-term follow-up of a previously published CBP® subject report with beneficial reported outcomes and objective measures stable over time.

## Case presentation

**Figure 1:**
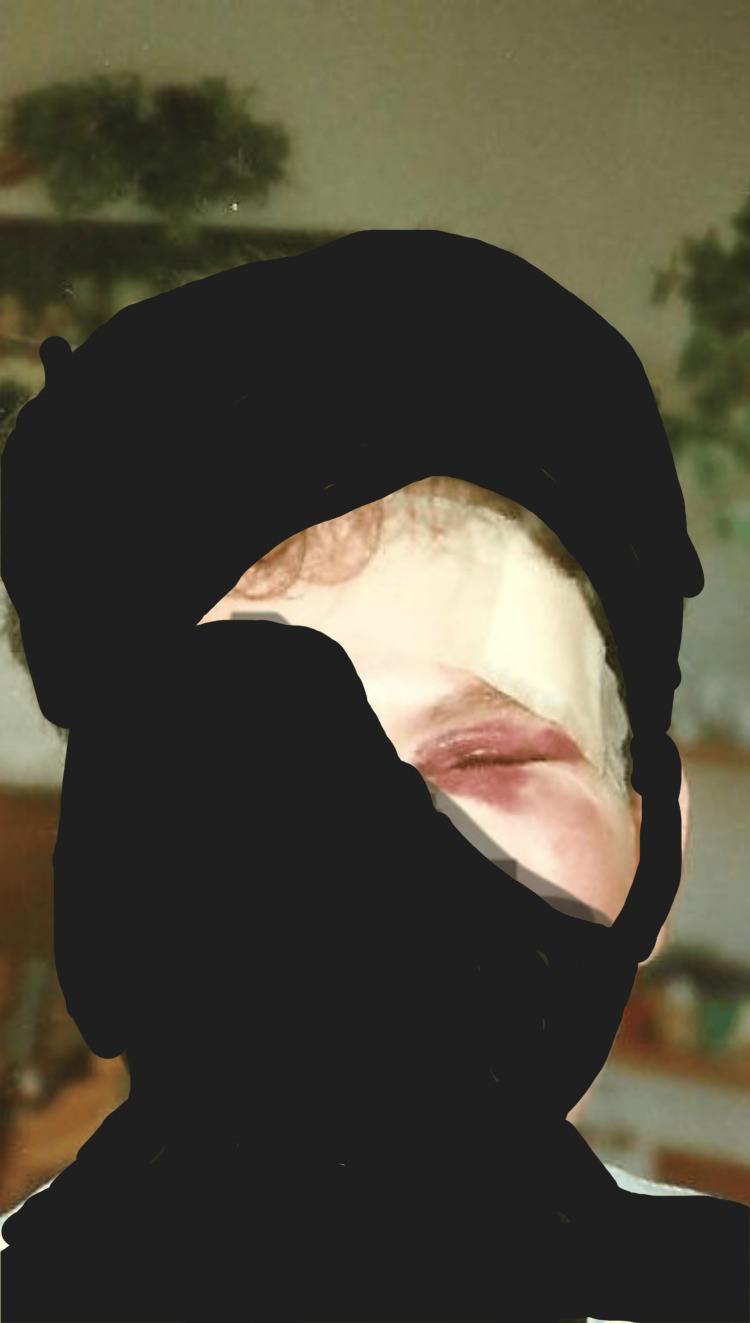
The patient approximately 72 hours after being struck by a hockey puck in the left eye and temple. The left orbit was fractured and there is significant soft tissue contusion and inflammation. The direction of the force of the puck was traveling from the patient’s left to right possibly contributing to or causing the significant head translation to the patient’s right and severe head pain.

In early 2010, a 55-year-old female patient presented to a spine rehabilitation facilitation in Montour Falls, NY, USA seeking treatment for NP and HAs, chronic neck stiffness, and abnormal posture. She reports a long history of spine trauma, including two cervical acceleration-deceleration (CAD) car crashes both causing injuries to the neck upper back, and shoulders as well as frequent chronic HAs that had been worsening for the prior four years. The HAs would be so severe that they would cause temple-pounding sensations and frequently lead to vomiting. They were made worse by allergies including dust and mold. She reports the second motor vehicle collision (MVC) caused her head to strike the windshield and the HAs and neck and shoulder pain increased significantly following that injury [10]. The patient also reports that approximately 11 months prior to the evaluation, she was struck in the face by a hockey puck at a professional game and the puck was likely traveling somewhere between 60-90 miles per hour which is average for NHL players. She was taken to the hospital and evaluated for her injuries. The impact caused an orbital fracture and required eleven sutures for the laceration. The physicians prescribed anti-inflammatories and pain relievers and discharged her without any future treatment recommendations. She had never suffered from NP or HAs prior to the injury. Her symptoms were worsening which led her to seek treatment (Figure 1) [11].

**Figure 2:**
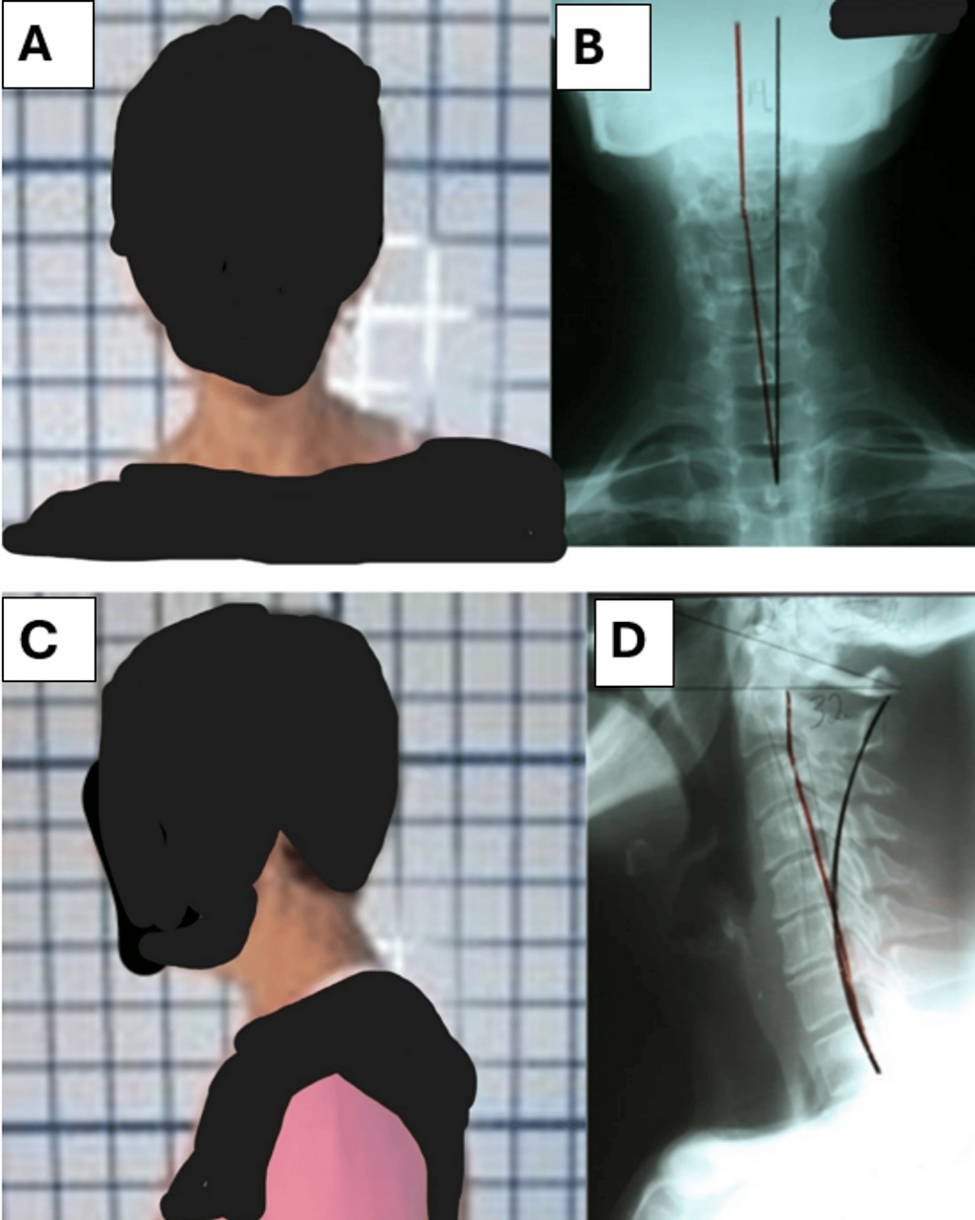
The image on the upper left (A) is the patient’s initial anterior-posterior postural image showing significant right head translation. The image on the upper right (B) is the anterior-posterior radiograph taken on the same day confirming the head translation. The image on the lower left (C) is the initial lateral posture image and the lower right (D) is lateral cervical radiograph demonstrating significant loss of normal lordosis.

An initial evaluation found a numerical pain rating scale (NPRS) (5/10; where 0 is normal and 10 is the worst pain ever) demonstrated moderate/severe NP. Her HAs she rated (9+/10) indicating severe, debilitating pain [12]. Cervical ROM testing for pain found restriction and pain with flexion, limited cervical extension, and an increase in pain from as little as fifteen degrees of extension. Bilateral lateral flexion as well as bilateral rotation were unremarkable. Shoulder abduction testing found reported pain. The patient’s neck disability index (NDI) was measured (6/100) indicating mild disability [13]. The postural analysis found right head lateral translation (-TxH), forward head posture (+TzH), slight left lateral flexion (-RzH), right thoracic translation (-TxT), slight thoracic flexion (+RxT), right posterior pelvic rotation (-RyP), and slight pelvic flexion (+RxP) (Figures 2A-2D).

Upright radiography of the spine was obtained according to all Medicare, state, and federal laws and guidelines by a physician with more than 10 years of experience. The spine parameter assessment was evaluated using PostureRay® radiographic analysis software (PostureCo Inc. Trinity, Fl, USA). Using the Harrison posterior tangent measurement for the sagittal spine and modified Risser-Ferguson evaluation of the anteroposterior (A-P) spine. These measurements were digitized and recorded. All spine parameters herein are reported as such: (Patient data #/ Normal value #). The A-P measurements found the right head translation (14mm/0mm). Sagittal evaluation of the cervical spine found loss of the normal lordosis (-16°/-34°) indicating significant hypolordosis as well as skull flexion measured with the atlas plane line (APL) demonstrating reduced normal position (-18°/28°). Degenerative changes at the levels with the greatest loss of lordosis between C3-C6 demonstrated loss of height and arthritic endplate loading changes. The type of postural MI® exercises, traction, and spinal manipulative therapy (SMT) was determined by the severity and magnitude of the abnormal spine alignment.

The patient’s lateral head translation was the largest postural deviation and was treated with MI® exercises, SMT in the opposite position, and a specific translation device for correction of abnormal lateral head postures (Berry Translation Traction Montour Falls, NY, USA) [14,15]. The prescribed protocol was used on the patient in-office three times per week for 12 weeks and re-assessment was performed following the treatment with at least 24 hours between the last traction treatment and the radiography and assessment. The patient was instructed to perform the exercises daily at home as well and was instructed to increase duration and intensity if symptoms did not worsen. Ergonomic and postural advice was provided to ensure that the patient was not worsening their condition with postures assumed during activities of daily living (ADLs). The patient performed in-office postural exercises consisting of actively contracting the muscles of the neck and upper back in the direction away from the abnormal posture. The standing MI® exercise was performed by the patient in the office to ensure proper positioning. The patient placed the left shoulder against a padded block on the wall and actively shifted the head in the opposite position of the abnormal posture. The duration of the contraction gradually increased, and the quantity of exercises increased over time to recruit and strengthen the neck musculature in the direction of normal posture.

**Figure 3:**
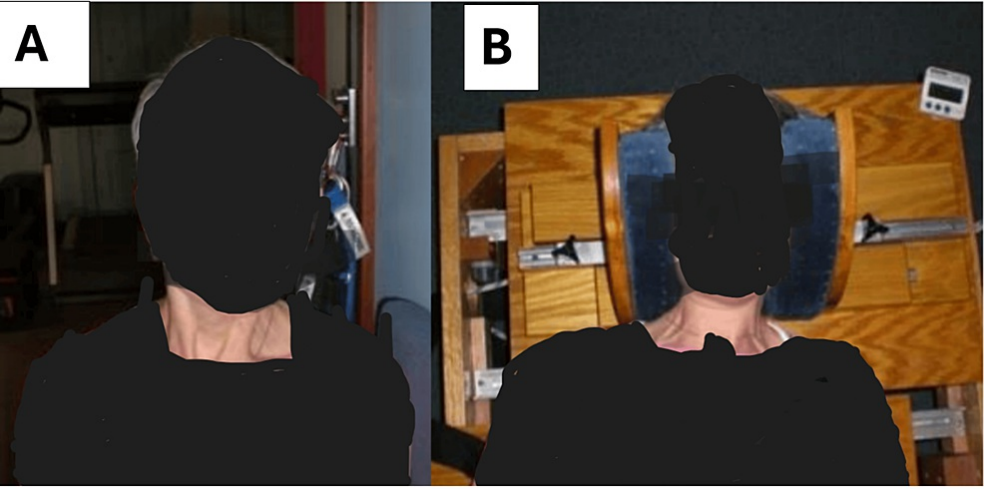
The image on the left (A) shows the patient performing the mirror image (MI®) exercise to strengthen the postural muscles and balance the head on the thorax. In the image on the right (B), the patient in the Berry translation traction where the thorax is stabilized and a mobile piece on the table translates the patient’s head and neck away from the abnormal posture in the MI®.

The Berry® translation traction device consists of a table with straps and movable segments to allow the patient to be supported and a lateral traction force is then applied in the specific direction that is the MI® of the abnormal posture and radiographic findings (Figures 3A, 3B) [14]. The patient begins in the traction device for 2-5 minutes and progresses in time and magnitude of the lateral shift in the MI® direction for up to 15 minutes. The physician ensured at each treatment that the patient was not experiencing an increase in symptoms due to the traction.

MI® postural SMT was performed by a chiropractic physician to increase ROM and reduce pain as well as improve the patient’s tolerance of traction and exercises. The patient was placed in a side-lying position and the adjustable part of the table’s headpiece was moved to induce translation in the MI® direction. An SMT force was applied to the cervical spine to assist in improving the patient’s posture. 

The patient was very consistent with both in-office treatments and home exercises. All informed consent and consent to publish were signed by the patient. All evaluations were performed by the same physician credential and under the guidance and complicated with all state, federal, and US Medicare laws. 

Results

**Figure 4:**
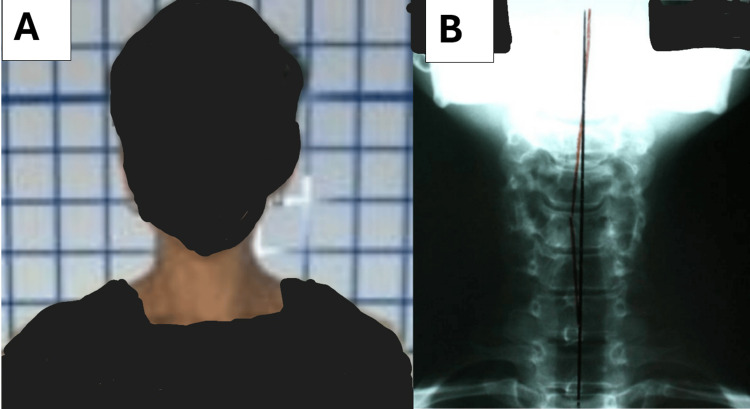
The post-treatment posture photo (A) and anterior-posterior cervical radiograph (B) both demonstrating near normal posture and spine position following the treatment protocol.

The same physician who performed the initial re-assessment found significant subjective and objective improvements following 1st re-assessment. NPRS for NP reduced significantly (1/10) and HAs had resolved (0/100). NDI demonstrated symptom resolution (0/100), and all ROM was found to be in full motion without any reported pain. The follow-up radiographs as well as the postural assessment demonstrated resolution of the lateral translation spine misalignment and the abnormal coronal balance. The patient had resumed all ADLs and was living her life pain-free and without any disability due to the previous HAs and NP. The subject was able to resume gym exercise and resumed running after several years, running up to ten miles per day and competing in races. The patient was pain-free and was exceptionally satisfied with her treatment. She continued to perform postural exercises daily (Figures 4A, 4B).

Thirteen years later, in August of 2023, the patient was recalled for a long-term follow-up of the previously reported data [14]. She received an evaluation including history since the last visit, orthopedic and neurological testing, dual-inclinometry for ROM and painful motion as well as spine radiography and postural assessment. Objective HRQoL measure was performed. She reported no new injuries, accidents, or reasons to exacerbate her prior NP and HAs. She reported having no NP or HAs causing any appreciable discomfort for several years since the original treatment. She reports being able to able to perform all ADLs without pain. Orthopedic and Neurologic testing was unremarkable with no definitive positive tests.

**Figure 5:**
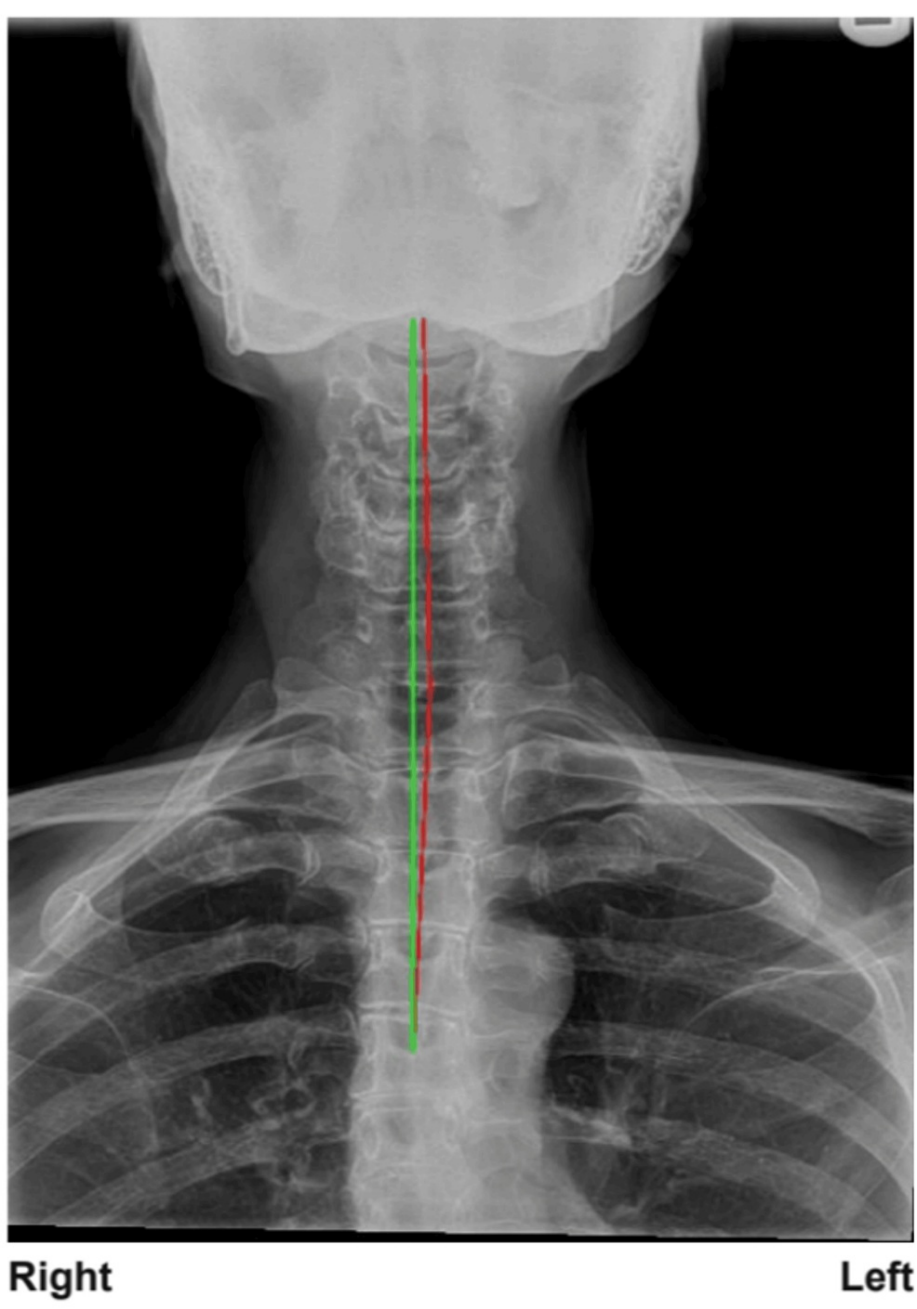
The 13-year follow-up anterior-posterior cervical radiograph demonstrating continued normal posture and spine alignment correlated with no return of any original complaints/symptoms. The measured head translation was less than 5 mm and within normal limits. The red line represents the centroid of each vertebra connected via a line and the green line is an ideal vertical axis line.

ROM to assess discomfort caused no reported pain. ROM measured via dual inclinometry found no restriction abnormalities compared to matched norms for sex and age; measuring cervical flexion (72°/50°), Cervical extension (30°/60°), left lateral cervical flexion (45°/45°), right lateral cervical flexion (49°/45). NDI was found to be without any disability due to NP (0/100). Upright spine radiography found the cervical spine had a measured lordosis (-18.3°/-34°) demonstrating that the patient had slightly regressed and had less lordosis over 13 years without treatment. APL was ideal (-29°/-29°). Anterior head translation (AHT) was slightly beyond average limits (15.8mm/0-15mm) (Table 1). Degenerative changes at prior levels (C3-C6) remained however did not appear to be a cause of discomfort or disability for the patient. She reported that she never suffers in the fashion she did prior to the initial treatment, and she continues to be as active as possible without interference, she is very grateful and thankful for her regained ability to function following structural rehabilitation 13 years prior (Figure 5).

**Table 1:** The initial, re-assessment, and 13-year follow-up results. NPRS-Numerical Pain Rating Scale, NDI-Neck Disability Index, ARA- Absolute Rotation Angle, Tz- Translation of the head on the z-axis, APL-Atlas Plane Line, Tx- Translation of the head on the z-axis

Evaluation	NPRS-Neck	NPRS-Head	NDI	ARA C2-C7\Ideal	Tz C2-C7\Average	APL\Ideal	T_x_ C2-C7\Ideal
Date							
10/12/2009	5\10	9\10	6%	29°\42°	32mm\0-15mm	27°\28°	14mm\0mm
2/44/2010	1\10	0\10	0%	32°\42°	14mm\0-15mm	27°\28°	0mm\0mm
8/23/2023	0\10	0\10	0%	18°\42°	15.8mm\0-15mm	29°\28°	2.3mm\0mm
Overall Change	5\10	9\10	6%	11°	16.2mm	2°	11.7mm

## Discussion

We present the 13-year follow-up of a previously reported case successfully treated with a multi-modal conservative treatment approach for a brief treatment period. A total of 36 in-office treatments, over 12 weeks, consisting of exercises designed to improve abnormal posture; sustained specific traction loads designed to improve abnormal spine alignment; and posture-specific SMT. The patient had symptom resolution following the initial 36 treatments continued to perform the home exercises and was able to maintain a healthy, athletic, pain-free lifestyle. Following a 13-year interim, a follow-up evaluation found continued posture and spine stability and a continued resolution of initial symptoms.

NP is extremely common in the global population and represents a substantial cause of disability due to musculoskeletal (MSK) disorders and a large contributor to GBD. NP is most frequently found in older females, those with a history of smoking, and high-stress work environments [16-19]. Prior trauma and a history of neck and spine pain and HAs significantly predispose individuals to report pain interference with ADLs as well as current and future risk of disability [19-21]. Women are more susceptible to injury and development of chronic pain from CAD trauma as was found in our patient. Buckling injury of the spine and the subsequent injury to the tissues increases inflammatory exudates, increases the fibrotic growth of scar tissues, increases nociceptive sensitization and the abnormal load will lead to degeneration. This degeneration and scar tissue make the patient more likely to suffer severe consequences from subsequent injuries as well as the sequelae associated with failure to heal from the previous trauma [22-25].

Individuals suffering from HAs and NP have a much greater number of years lived with disability than non-HA and non-NP populations and considerable resources are spent every year to treat HA and NP patients globally [26-28]. Treatment options for HA and NP and injury-related chronic spine pain from whiplash-associated disorders (WAD) range from very conservative over-the-counter (OTC) medications such as NSAIDS to prescription medications, physical therapy, radiofrequency nerve ablation, steroid, analgesic, and other types of joint injections, as well as minor and major surgical procedures [29-36]. Successful treatment demonstrating both subjective and objective improvements in PROs and HRQoL as well as improved sagittal and coronal balance with better spine alignment stable at long-term multi-year follow-ups are extremely rare and complications are reported frequently due to failed treatment, tissue damage, and altered loads [37-40].

Treatment with CBP® protocols has shown growing evidence of improvement in spine and postural balance as well as improvements both short- and long-term in HRQoLs as well as objective radiological assessment of posture and spine alignment. This treatment involves a multi-modal regimen designed to strengthen weakened postural muscles and improve spine loading via postural MI® traction, as well as gentle postural and intersegmental SMT. The treatment is designed to alleviate pain from the initial injury, followed by a program of increasing intensity to improve muscular and spine tissue strength and endurance, as well as stabilize the spine toward normal upright posture with improved neuromuscular balance. CBP® methods have been confirmed at the case report and case series level, via cohort studies, biomechanical and modeling investigations, reliability and repeatability studies as well as randomized controlled trials (RCTs) and systematic reviews of the literature with positive results [1-5,41-47].

Upright radiography was used in this subject’s diagnosis and treatment and the simple use of upright spine radiographs continues to be the criterion standard in spine care and treatment. Without diagnostic imaging, physicians treating spine pain and disability are at much greater risk of missing differential diagnosis, complications such as unexpected degeneration and instability, as well as morphological, genetic, and previous trauma contributions that may alter diagnosis and treatment options significantly. Likewise, measurement using machine learning programs such as PostureRay® gives astute clinicians a tool to determine the success of their treatments to reduce coronal and sagittal imbalance as well as specific intersegmental and regional spine misalignment [47-53]. Limitations of this study are the single patient size as this is too small to make definitive or absolute conclusions. An additional limitation is the use of a multi-modal treatment regimen without the certainty of the influence of each intervention on overall improvement. Additionally, the patient's success conclusions would have been more robust with an assessment of additional HRQoLs.

## Conclusions

We presented short-term improvements in a patient suffering for many years prior to treatment with NP and HAs, muscular stiffness, and disability. The subject improved with MI® traction, exercises, and SMT. Outcomes following treatment, including post-treatment, and recent longer-term follow-up found stability of the subjective and objective outcome measures.

NP and HAs will remain a significant source of suffering and disability globally and will contribute to GBD as well as reduce HRQoL and impact billions of humans yearly. Safe, simple to apply, economical interventions should be sought by clinicians who treat individuals suffering from spine conditions and disability related to chronic trauma injuries and CAD. This case report with very long-term follow-up contributes to a growing body of evidence demonstrating the efficacy, repeatability, and validation of these methods across many conditions and populations. Further, larger studies are necessary to make firm conclusions regarding efficacy to determine if CBP® reduces GBD.
